# How ICT can contribute to realize a sustainable society in the future: a CGE approach

**DOI:** 10.1007/s10668-021-01674-9

**Published:** 2021-08-05

**Authors:** Xiaoxi Zhang, Machiko Shinozuka, Yuriko Tanaka, Yuko Kanamori, Toshihiko Masui

**Affiliations:** 1 Space Environment and Energy Laboratories, NTT, 3-9-11, Midori-Cho, Musashino-Shi, Tokyo 180-8585 Japan; 2 NTT Advanced Technology Corporation, 3-9-11, Midori-Cho, Musashino-Shi, Tokyo 180-8585 Japan; 3 National Institute for Environmental Studies (NIES), 16-2, Onogawa, Tsukuba, Ibaraki 305-8506 Japan

**Keywords:** ICT, Environmental impacts, GHG emissions, GDP, Scenarios, CGE model, Digital technologies

## Abstract

Many information and communications technology (ICT) services have become commonplace worldwide and are certain to continue to spread faster than before, particularly along with the commercialization of 5G and movement restrictions in response to the COVID-19 Pandemic. Although there is a concern that ICT equipment usage may increase power consumption and emit greenhouse gas (GHG) emissions, ICT has also been contributing to reducing GHG emissions through improved productivity and reduced mobility. This research targeted the main ICT services used in Japan and adopted a dynamic national computable general equilibrium model to quantitatively analyze future impacts on economic growth and GHG emission reduction until 2030 by using these ICTs, while considering both the increase in power consumption of ICT itself and the reduction in environmental load in other sectors. The results showed that the spread of ICT services, especially some artificial intelligence-based services, can improve productivity in most sectors through labor-saving and contribute to improving overall gross domestic product (GDP). Additionally, increased efficiency of logistics and manufacturing can greatly reduce the input of oil and coal products and so greatly contribute to GHG emission reduction. In 2030, compared with the baseline scenario in which all technology levels are fixed at current levels, at least 1% additional GDP growth and 4% GHG emission reduction can be expected by the targeted introduction of ICT in the ICT accelerated scenario in which the technology level of ICT accelerates. This also means ICT can potentially decouple the economy from the environment.

## Background and research issues

Over the last two decades, great progress has been made in information and communications technology (ICT). Industrial ICT and daily online ICT services have become commonplace worldwide. ICT, as an industry, has already and will continue to have a major impact on economic and societal activities, such as GDP growth, employment, productivity, and quality of life (Palvia, 2017). ICT is also greatly contributing to improvements in productivity across industry (Oulton, 2002).

ICT has not only contributed to economic growth and convenience of life but also reduced environmental impacts. ICT can be used to make our patterns of production and consumption more sustainable (Hilty and Aebischer, 2014). For example, ICT enables more of us to work from home, thus reducing travel-related GHG emissions. Many sharing services reduce raw material use. ICT improves production efficiency and reduces the input of intermediate raw materials. The Global e-Sustainability Initiative estimated that ICT could generate over US$11 trillion in economic benefits per year by 2030; at the same time, ICT use could also enable the world to cut its global emissions by 12,000 Mt-CO2eq across 8 fields including smart buildings, smart mobility, etc. (GeSI, 2015). These can be considered as positive effects of ICT use on the environment. Therefore, ICT could also be considered as a GHG emission reduction measure to tackle the global issue of this century, climate change (IPCC, 2014).

However, technological developments such as AI, IoT, 4G, and future commercialization of 5G have brought about a large increase in communication traffic data. The reasons for this increase include a higher numbers of Internet users, more devices and connections, faster broadband speeds, and increased video viewing (Barnett, 2018). Increases in energy consumption and GHG emissions attributed to the ICT sector, which have a negative effect on the environment, are becoming an increasing concern. The International Telecommunication Union Telecommunication Standardization Sector (ITU-T) reported the baseline GHG emissions of global ICT sector development in 2030 will be 1.3 times that in 2015, without considering developments in the power sector (L.1470, 2020). Another study also estimated that in the worst-case scenario, the global electricity usage of ICT could contribute up to 23% of global GHG emissions by 2030 (Anders, 2015).

Economic recovery and GHG reduction are both important, especially in the post-pandemic era. The question for us is while ICT boosts economic growth, whether or not the positive effects of ICT’s increasing permeation into various sectors of the economy will outweigh negative effects associated with increased power consumption. In other words, can we use ICT sustainably to decouple economic growth and its environmental impacts?

To answer the above question, it is very important to grasp the overall future environmental and economic impacts of ICT use including positive and negative effects. Methods have been proposed to assess the positive and/or negative effects of ICT use (GeSI, 2015; Pohl, 2019; Moyer, 2012; L.1410, 2014; L.1451, 2019; Erdmann, 2010; Bastida, 2019; Nagao, 2017). Taking a bottom-up approach for individual ICT services, Recommendation L.1410 published by ITU-T proposed a method for the environmental lifecycle assessment (LCA) of ICT goods, networks, and services (L.1410, 2014). The method can well understand CO_2_ emissions from each life stage of ICT use. However, it is more suitable for specifically analyzing individual ICT, since it requires many detailed micro-level data, and it has difficulty grasping the whole effects at a country level or for future forecasts. Researchers at Accenture used a more macro-method based on 12 ICT use cases (such as “E-health”) (Bieser, 2018). They estimated the level of ICT adoption and the impacts on GHG emission of ICT adoption in each use case and compared them with the baseline. Furthermore, there are effects of ICT use that are more complex, such as rebound effects and spillover effects across industries (Lorenz, 2014). Rebound effects negatively affect the intended positive effects (Bomhof, 2009). Several studies have mentioned that CO_2_ emission reduction may rebound depending on the types of individual ICT (Gossart, 2015; James, 2019; Galvin, 2015; Takahashi, 2004). However, the above methods have difficulty covering all these impacts completely, especially when considering the future impact. To grasp the overall impacts at a macro-level, including the complex effects, computable general equilibrium (CGE) analysis as a top-down approach is more suitable than the above bottom-up approach. It can help us grasp the economic and industrial structural changes caused by the technologies spreading not only at present but also in the future and understand the impacts on not only the economy but also the environment. CGE analysis is usually used for analyzing the effects of general economic policies and various energy and climate policies. For example, several studies have analyzed GHG emission reduction effects and economic effects of environmental measures, such as introducing carbon taxes and expanding use of renewable energy and cleaner technologies (Chunark, 2017; Boonpanya, 2021; Matsumoto, 2011; Oshiro, 2017). However, few studies have analyzed the environmental effects of ICT usage from this approach, due to the complexity of expressing and assessing the effects of ICT introduction in the CGE model and the large amount of actual effect data required. Our previous study combined a bottom-up approach (mainly based on the LCA method) and a top-down approach (CGE analysis method) (Zhang, 2020). In the previous study, the direct effects of introducing ICT services are investigated and estimated mainly on the basis of the LCA approach and are expressed in the CGE model. The authors developed a dynamic CGE model to forecast the future macroeconomic and environmental impacts until 2030 by using some of the main ICT services in Japan. However, it focused mainly on the positive effects caused by ICT usage but hardly considered the increases in Internet traffic and changes in energy consumption caused by using ICT equipment, which is also a big concern for the future as mentioned above.

The purpose of this research is to quantitatively analyze future overall impacts of ICT usage in Japan on the economy and environment, considering both positive and negative effects on the environment, respectively. This will allow us to clarify whether economic growth from ICT can be decoupled from its and environment impacts. This will enable ICT to be used more sustainably and help to design policies that encourage environmentally advantageous utilization of ICT, while inhibiting uses that increase the speed of GHG emissions and resource consumption.

## Estimation method

### Basic structure of the CGE model

**Fig. 1:**
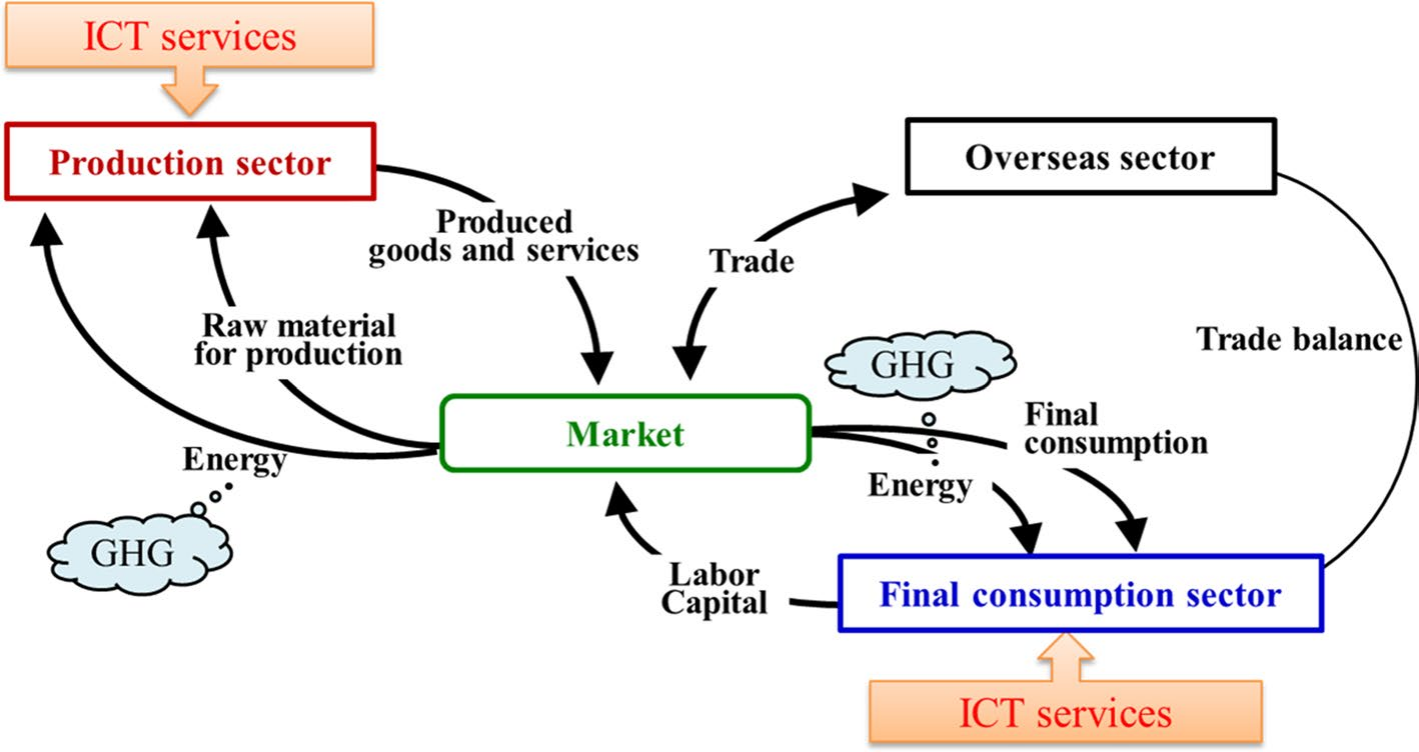
Structure of CGE model

The model used in this research is based on the dynamic national CGE model for Japan, Asia–Pacific Integrated Model/Computable General Equilibrium (AIM/CGE [Japan]) (Masui, 2003). Its advantage is that it can treat different technologies including ICTs, so the model can analyze the impacts on economic and environment aspects by introducing these technologies. The CGE model in this research is calibrated with the 2005 Input–Output (IO) Tables in Japan (MIC, 2009). It has 49 sectors and 43 commodities, and electricity production is disaggregated into 10 sectors by power generation technologies as shown in “Appendix 1.1”. Figure 1 shows the basic structure of the CGE model. The model contains four blocks: production sector, final consumption sector, market, and overseas sector.

#### Production sector and final consumption sector

The production sector uses production factors (e.g., capital and labor) and intermediate inputs (e.g., energy and raw materials) to produce products and supply them to the market. The production sector conducts production activities to maximize their profits subject to the availability of their production technologies. The final consumption sector holds the production factors, provides them to the production sector, and receives income as pay. Utility is calculated from the final consumption using the Cobb–Douglas function, and the purchased amount of consumption is determined to maximize the utility, subject to the constraints of budgets and commodity prices. Appendix 1.1 details the structure for production activities and final consumption.

#### Market equilibrium condition for the CGE model

Under the economic balance, a price mechanism determines levels of activity in each sector and prices for all goods, services, and production factors. That means that if demand exceeds supply, then prices will rise. Conversely, if demand is less than supply, prices will fall. The model’s equations also include a set of constraints that have to be satisfied by the system as a whole. The key equations used in the model are listed in Appendix 1.1 and can be referred to (Masui, 2003).

#### Overseas sector

The production is distributed for export and domestic use. Here, a constant elasticity of transformation (CET) function with constant deformation elasticity is assumed. The domestic markets are integrated with domestic goods and imported goods by the constant elasticity of substitution (CES) function.

#### Environment loads treated in the model

The main environmental load treated in the model is GHG emissions. GHG emissions from each sector in the model in the base year are reconstructed on the basis of GHG emission data estimated by the Greenhouse Gas Inventory Office of Japan (GIO, 2019) and are adjusted to reproduce Japan’s total GHG emissions. The emissions from energy sources are calculated in accordance with the amount of energy consumed in each sector, and other emissions are calculated in proportion to the amount of activities in each sector. Furthermore, in addition to GHG emissions, this research also briefly analyzes the impacts on raw material consumption, including resource use.

### Introduction of ICTs

When production sectors and the final consumption sector introduce ICTs, demand from these sectors for ICT services will increase, which will bring about positive effects. For example, ICT usage is expected to increase production efficiency in the production sector and consumption efficiency in the final consumption sector, which means the intermediate inputs required per unit of production will be reduced. Production automation will save more labor. Using supply and demand matching technologies will reduce loss and waste and so reduce production. In this research, the authors investigated a large amount of statistical data and related reports (e.g., MICa, 2019; NEDO, 2016) and estimated these kinds of direct effects on production and consumption activities caused by introducing ICTs and expressed the direct effects by changing the IO coefficients in corresponding sectors in the CGE model. Due to these changes, the related market will be temporarily out of equilibrium. The CGE model can calculate to balance the supply and demand in each market on the basis of a price mechanism for each good and production factor.

### Dynamic progress

**Fig. 2:**
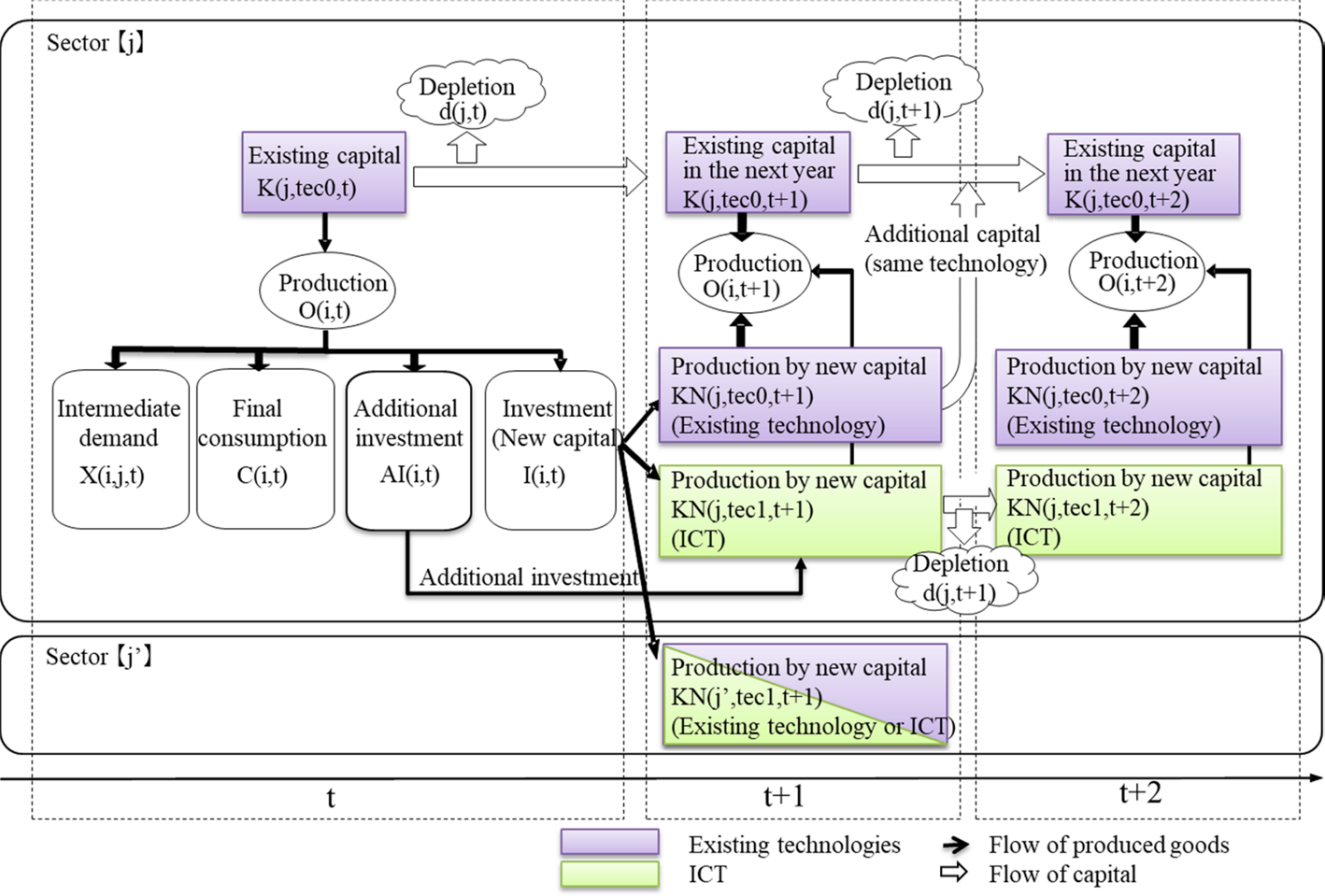
Process of capital accumulation in the dynamic CGE model

For dynamic progress, as mentioned in the previous section, the model parameters are calibrated to replicate the IO Tables in the base year, 2005 (MIC, 2009). Figure 2 shows the process of capital accumulation and productions in our dynamic CGE model. Equilibrium calculation starts from 2005. Productions in sector j are used for intermediate demand, final demand, investment, and additional investment for introducing ICTs. The investment can be used as new capital for not only the existing technology and ICT in sector j but also other sectors j^’^ in the following year, 2006. To maximize profit in the equilibrium calculation, the model endogenously decides the allocation of the new capital. However, in this model, once the new capital is introduced into one sector, it cannot be moved. Existing capital stock in sector j will be used for the same sector in the following year taking depreciation into account. Then, capital stock in 2006 is determined by existing capital stock in the previous year (2005), depreciation rate, and investment from 2005. Depending on the technology levels of existing capital stock and new capital investment, the model calculates efficiency levels in each sector in 2006, such as energy efficiency. Moreover, the equilibrium solution in 2006 will be calculated on the basis of the prepared efficiency levels. The model will calculate the equilibrium solution year by year.

In this model, the ICT services will be introduced by allocating the new capital for ICT services. That is to say, the retrofitting of ICT services to existing capital is not assumed in this model. New capital is provided to ICT services year by year, and expansion in the share of new capital provided to ICT services expresses the spread of ICT services. The amount of ICT introduced determines the additional investment for ICT endogenously in the model.

### Input data and future baseline scenario

The CGE model calibrated the necessary parameters using the IO Table in Japan in 2005 (MIC, 2009). For a future scenario from 2016 to 2030, Table 1 shows basic preconditions, such as population changes, expected gross domestic product (GDP) growth rate, and change in power supply configuration, which are set on the basis of Japan Intended Nationally Determined Contributions (MOFA, 2015).

**Table 1:** Preconditions in future baseline scenario

		2005 year	2015 year	2030 year (estimation)
Population		127.8 million	126.6 million	116.6 million
Households		49.0 million	53.4 million	54.7 million
GDP growth rate		1.2%/year	0.9%/year	1.7%/year
Power supply configuration	Renewable	8%	13%	22 ~ 24%
	Nuclear	31%	1%	20 ~ 22%
	LNG	23%	41%	27%
	Coal	27%	35%	26%
	Oil	11%	10%	3%

## Target ICT services and future ICT scenarios

### Target ICT services

The paper targeted 36 kinds of ICT services used in six industry categories: finance, public services, manufacturing, distribution and services, medical and agriculture, and infrastructure. All 36 kinds of ICT services are divided into two groups. Group 1 includes ICT services that are already common, such as teleworking and online shopping. These ICT services are already maturing and have relatively high utilization rates according to statistical data in Japan (MICa, 2019). Group 2 contains relatively new ICT services, such as AI and IoT. Even though these ICT services have not spread as widely as the first group of ICT services in Japan, they have already started to be used in some industries, like manufacturing and distribution and services, and are expected to greatly reduce GHG emissions in the near future. Table 2 lists ICT services of Groups 1 and 2. The shaded cells represent the ICT services in Group 2, and the others represent the ICT services in Group 1.

**Table 2:** Targeted ICT services, direct effects expected, and future scenarios for ICT services

Industry category	ICT services	Effect expected by ICT usage	2030 scenarios		
			Index of utilization	Baseline (keep in 2015 level)	ICT accelerated
(A) Finance	A1 Online banking	Reduction of transportation use; reduction of branch banks	Online banking accounts	82.09 million accounts	146.46 million accounts
	A2 Electronic bonds	Reduction of physically transporting bonds; reduction of operation	Electronic bond records	5.82 million times	23.71 million times
	*A3 Cashless payment*	*Cost reduction in management of store business; cost reduction in operation and management of ATMs*	*Utilization rate*	*0.00%*	*50.00%*
	*A4 Digital technology in banking*	*Reduction in office work and customer service in banking*	*Utilization rate*	*0.00%*	*50.00%*
(B) Public service	B1 Electronic bidding	Reduction of transportation use	E-biddings	83,000 times	960,000 times
(C) Manufacturing	C1 Supply chain management	Suppression of overproduction; optimization of intermediate distribution and retail sales; reduction of factory and storage space	Utilization rate	40.00%	54.00%
	C2 Matching service for re-use of used-car parts	Reduction of resource use; reduction of office use; reduction of waste	Recycle rate × B2B EC rate	0.29%	0.45%
	C3 Matching service for re-use of industrial machinery		Recycle rate × B2B EC rate	1.69%	2.68%
	C4 Matching service for re-use of construction machinery		Recycle rate × B2B EC rate	1.43%	2.27%
	C5 Matching service for re-use of computers		Recycle rate × B2B EC rate	2.16%	3.43%
	*C6 IoT technology for manufacturing*	*Productivity improvement by visual control in production line; reduction of lead time*	*Utilization rate*	*0.00%*	*50.00%*
	*C7 AI technology for manufacturing*	*Productivity improvement by using machine to set up or inspect equipment instead of skilled technicians; improvement of operation rate; prevention of human error*	*Utilization rate*	*0.00%*	*50.00%*
	*C8 Industrial robots*	*Productivity improvement*	*Utilization rate*	*0.00%*	*50.00%*
	*C9 Electronic procurement*	*Cost reduction on operation*	*Utilization rate*	*0.00%*	*50.00%*
(D) Distribution and services	D1 B2C e-commerce (EC)	Optimization of intermediate distribution and retail sales; increase of inventory space at net retailers and goods distributed by parcel delivery; suppression of overproduction; reduction in volume of returned goods	EC rate	5.79%	12.20%
	D2 Online issuing of air tickets	Reduction of transportation use	Rate of online reservation	52.00%	72.00%
	D3 Purchase of tickets at convenience stores		Utilization rate	18.40%	25.40%
	D4 B2B e-commerce (EC)	Reduction of transportation use; optimization of accounting works, intermediate distribution, wholesale, and retail sales	EC rate	29.60%	41.30%
	D5 Online music service	Optimization of intermediate distribution and retail sales; unnecessity of storage space and retail shops; reduction in producing CDs/DVDs/ newspapers and books; reduction in sales distribution and returned goods delivery	Online utilization rate	53.60%	53.60%
	D6 Online video service			48.50%	60.00%
	D7 Online PC software			37.00%	100.00%
	D8 Digital books		E-book utilization rate	13.92%	47.90%
	D9 Remote management of vending machine	Reduction of transportation use	Utilization rate	34.00%	100.00%
	*D10 AI technology for demand forecast of food products in retail business*	*Reduction of food loss by advanced demand forecast*	*Utilization rate*	*0.00%*	*50.00%*
	*D11 AI technology for unmanned stores*	*Labor-saving by unmanned operation in stores*	*Utilization rate*	*0.00%*	*10.00%*
	*D12 AI technology for distribution*	*Productivity improvement in physical distribution*	*Utilization rate*	*0.00%*	*50.00%*
	*D13 AI technology for self-driving*	*Cost reduction due to the decrease in motor vehicle accidents*	*Utilization rate*	*0.00%*	*50.00%*
(E) Medical and agriculture	E1 Electronic medical records	Reduction of paper	Utilization rate	34.40%	74.40%
	*E2 Electronic prescription*	*Reduction of paper*	*Utilization rate*	*0.00%*	*100.00%*
	*E3 Electronic medicine notebook*		*Utilization rate*	*0%*	*100.00%*
	*E4 Smart agriculture*	*Improvement of efficiency on farm work by visual control based on sensor data; labor-saving by introduction of robots*	*Utilization rate*	*0.00%*	*50.00%*
(F) Infrastructure	*F1 AI technology for electricity demand forecast*	*Improvement of generating efficiency by advanced electric power demand forecast*	*Utilization rate*	*0.00%*	*100.00%*
	*F2 Smart meter for water supply*	*Labor-saving on metering work*	*Utilization rate*	*0.00%*	*50.00%*
	*F3 Smart house*	*Reduction of electricity consumption*	*Utilization rate*	*0.00%*	*100.00%*
(M) Both category (C) and (D)	M1 Teleworking	Reduction of transportation use; reduction of office use	Percentage of teleworkers	14.70%	34.00%
	M2 TV conferencing	Reduction of transportation use	Utilization rate	13.64%	29.00%

Italic values represent the ICT services in Group 2, and the others represent ICT services in Group 1.

### Direct effects expected by ICT introduction

Direct effects caused by ICT introduction (such as reduction of movement and materials and optimization of intermediate distribution and retail sales) are set the same as those in a previous study (Zhang, 2020) and are also listed in Table 2. For Group 1, since the services are already in common use, many data can be collected from official statistics (e.g., MICa, 2019), and direct effects are mostly estimated on the basis of these statistical data. For Group 2, direct effects expected by using these ICTs are surveyed by forecast reports for future ICT and some use cases that have already been introduced in manufacturing factories and banking (Nikkei BP, 2016; NEDO, 2016). The values of the direct effects are estimated by the total amount of the evaluated activity and the amount reduced by ICT use and will be fed back to the intermediate input coefficient of the CGE model.

### Future ICT scenario

To evaluate the impacts by using ICT, there are two main scenarios: baseline and ICT accelerated. Table 2 lists their details. For both scenarios, input data between 2005 and 2015 are set the same and are based on statistical data. To clarify the effectiveness of the ICT services independently, in the baseline scenario, energy efficiency for all technologies from 2016 to 2030 is assumed to be fixed at the level in 2015. In the ICT accelerated scenario, changes in ICT utilization rates and direct effects are set until 2030. Regarding the utilization rates, for Group 1, most data have been collected from related latest statistical data from 2008 to 2018 (e.g., MICa, 2019). The past data on utilization rates were fitted with the most fitted function and extrapolated until 2030. It can be seen that the utilization rates are quite different even in the same group. Less than half of the ICTs have utilization rates higher than 50. Therefore, the utilization rates of ICT technology can be believed to still have a lot of room for improvement and can be reasonably predicted to continue to along the increasing trend of past years. For example, the utilization rate of telework is predicted to be 34% in 2030 in this paper, but, due to the impacts of the COVID-19 Pandemic, the utilization rate of telework in Japan has already reached 28% in April according to data from 2020 (PERSOL, 2020). Then, for the emerging ICT services in Group 2, our main references are a forecast report on future ICT development in related industries published by Nikkei BP and a forecast report on AI technologies issued by NEDO (NEDO, 2016; Nikkei, 2016). They forecasted the kinds of emerging ICTs that are planned to be implemented in industry sectors and our lives and also the kinds of effects that can be expected by spreading these ICTs. AI technologies are expected to be widely implemented in four fields: manufacturing, mobility, medical/health, and distribution/retail and logistics. Additionally, cashless payment, smart agriculture, smart houses, and smart infrastructure are also highly expected to spread widely. However, since there are no detailed predictions of their utilization rates, the utilization rates of the targeted ICT services in this group are boldly assumed to spread to high levels in our model. Also, another reason is that this paper aims to show the potential impacts of ICT use. For most services in Group 2, utilization rates are assumed to reach 50% maximum, while for several services considered to spread easily, such as electronic prescriptions and smart houses, utilization rates are assumed to reach 100%. Additionally, the spread of AI technology for distribution is not a strong prospect, since it strongly depends on automated driving.

Investment values to ICT sectors between 2005 and 2020, such as equipment investment or software development, come from a market survey report about the ICT-related market (FCR, 2018), and the values until 2030 are estimated by linear approximation on the basis of the existing data.

### Power consumption forecast in ICT sector

To support these future ICT services, a lot of ICT equipment will be needed like sensors for collecting the data, edge computers, data servers, and 5G mobile network equipment. Thus, the increase in power consumption by ICT equipment is a big concern. In this research, the authors also investigated and analyzed power consumption trends of ICT equipment use in the ICT sector in Japan up to 2030 on the basis of data between 2008 and 2018 and related reports on market trend forecasts (e.g., JEITA, 2016). The ICT sector includes fixed/mobile networks, data centers, and end-user devices. To grasp the power consumption trends of the ICT sector itself, the authors attempted to estimate the total power consumption of ICT equipment until 2030 by estimating the changes in the number of each kind of ICT equipment and the changes in the power consumption per unit of each kind of equipment. “Appendix 1.2” shows more details of calculation conditions and data.

**Fig. 3:**
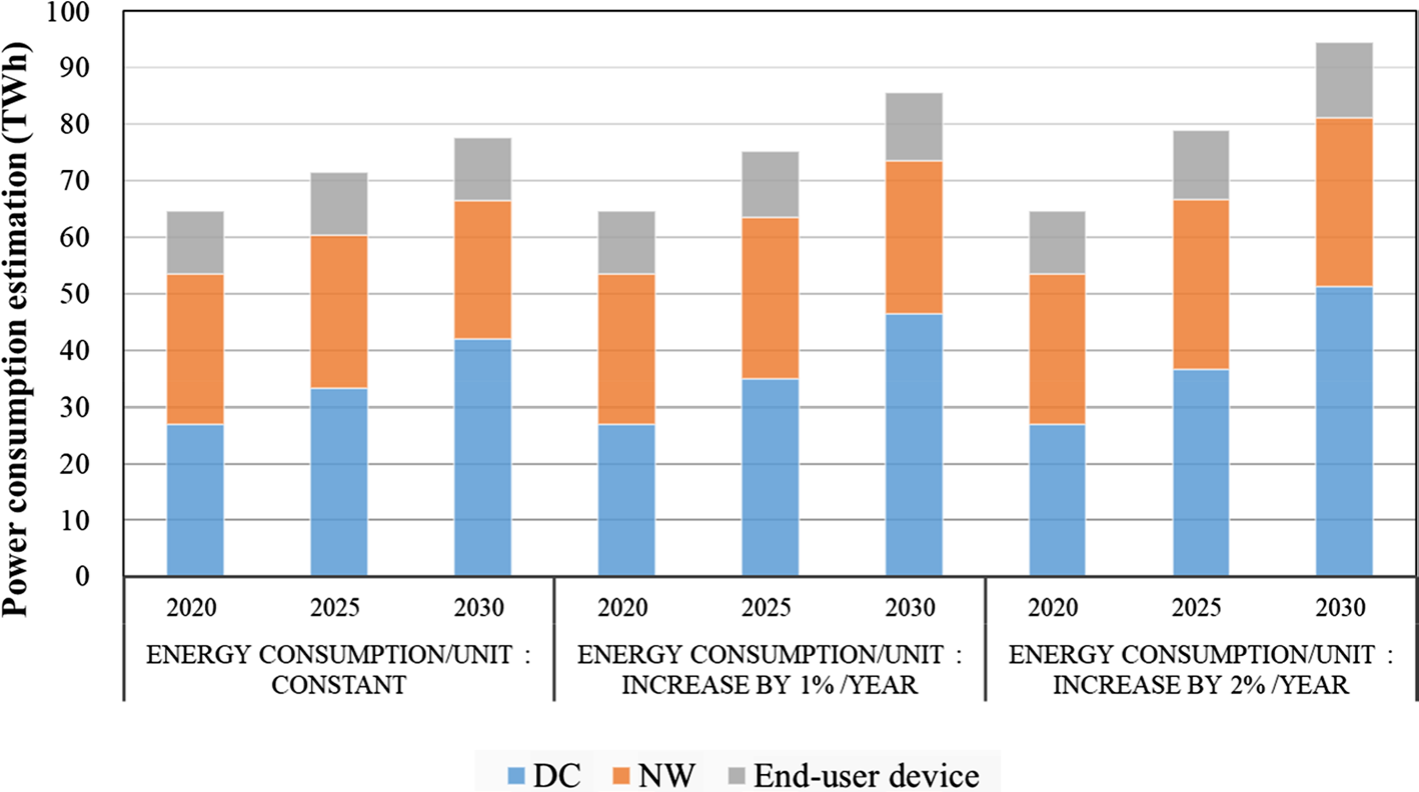
Estimation results on power consumption of ICT equipment by 2030

According to the three different assumptions on the increased rate of power consumption for each equipment unit per year, the estimation results showed that power consumption of total ICT equipment use in 2030 will increase to 1.2 to 1.5 times that in 2020 (Fig. 3). Since the number of ICT equipment does not change significantly, the change in energy consumption of each ICT equipment will play a decisive role in the total energy consumption in the next 10 years. If it is assumed that the energy consumption of each device increases by 2% per year, then the ICT total energy consumption in 2030 will be 1.5 times that in 2020. In the CGE model, service demands for ICT in non-ICT are adopted as an indicator to express the energy consumption increases or communication traffic increases in the ICT sector. From the viewpoint of changes in the demand for ICT in each sector, including a sensitivity analysis, the input coefficients of telecommunications services in each sector were set to increase up to 1.25, 1.50, and 1.75 times the default values.

## Results and discussion

### Results in each scenario

There are two scenarios set in this research: baseline, in which all technologies maintain the level of 2015 until 2030, and ICT accelerated, in which only ICT technologies accelerate in accordance with our forecast. In the ICT accelerated scenario, to give a sensitivity analysis of service demand increase for the ICT sector, the model simulated four cases: ICT_1.00, ICT_1.25, ICT_1.50, and ICT_1.75. That means service demand for the ICT sector from non-ICT sectors in 2030 is assumed to be 1.00 (the same as that in 2015), 1.25, 1.50, and 1.75 times that in 2015.

**Fig. 4:**
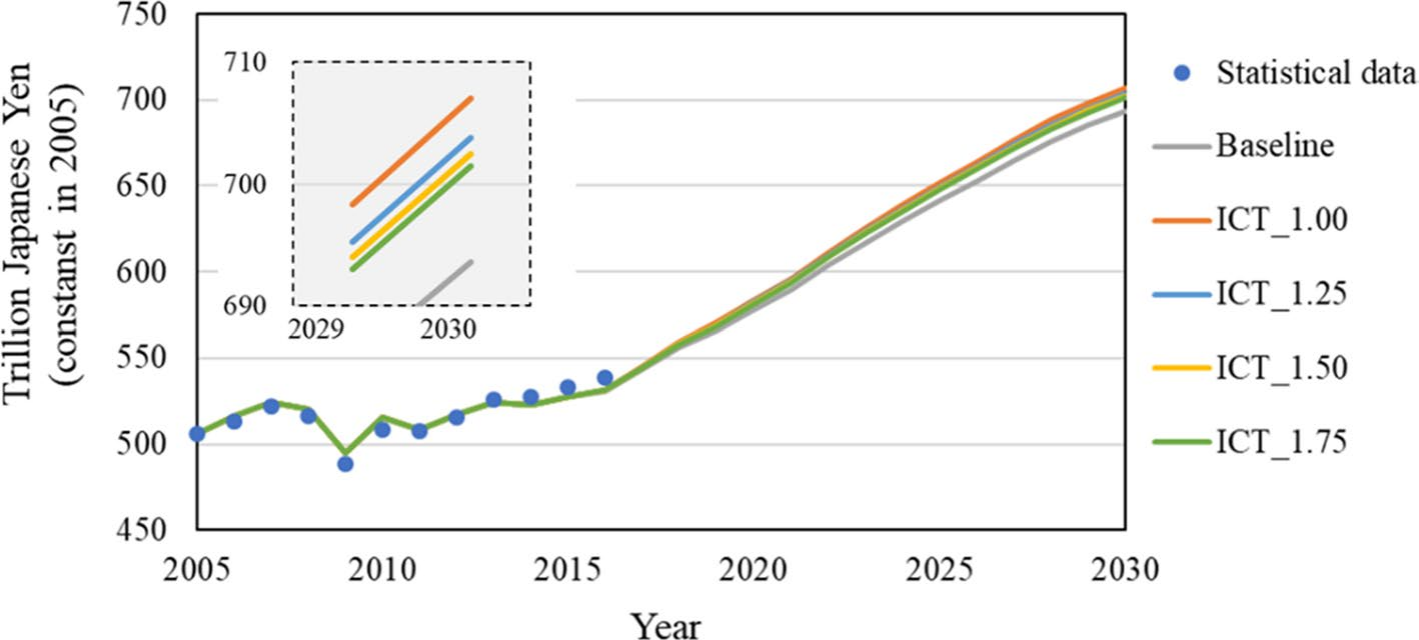
GDP during 2005 to 2030 in each scenario

First, Fig. 4 shows increases in GDP in each scenario. Since the pre-condition of GDP growth is assumed as 1.7% per year, in the baseline scenario, GDP will increase to about ¥700 trillion in 2030. In ICT_1.00, where ICT is introduced without any additional ICT service inputs, GDP increases 1.9% more, ¥13 trillion, compared with the baseline scenario. In addition, in ICT_1.25, ICT_1.50, and ICT_1.75, where the service demand for the ICT sector increases by 1.25, 1.50, and 1.75 times, GDP increases by 1.5% 1.3%, and 1.1%, respectively, compared with the baseline. Compared with the case where the increase in service demand for ICT is not taken into consideration, GDP growth tends to slow down. Nevertheless, an increase of 1% or more can be expected.

**Fig. 5:**
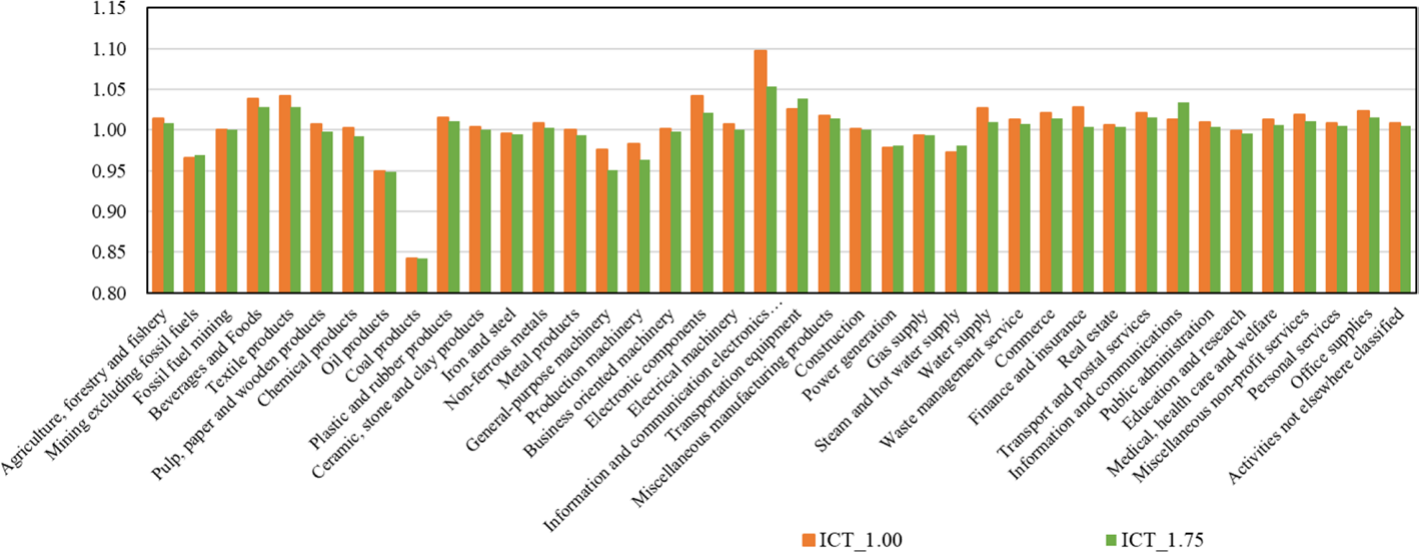
Production amounts by sector in two cases in 2030 (value in the baseline in 2030 = 1)

For more details, Fig. 5 shows the production amounts by sectors in 2030 in two cases, ICT_1.00 and ICT_1.75 in the ICT accelerated scenario, where the production amounts by sector in the baseline in 2030 are assumed to be 1. Compared with the baseline scenario, in each case of the ICT accelerated scenario, with the spread of ICT, the activities of the information and communication sector and information and communication electronics equipment sector will increase. Then, focusing on most service sectors, activities become more dynamic due to production efficiency improvements by ICT introduction, especially due to labor-saving and better matching between supply and demand. Also, as the total GDP grows, so do income levels of households. Then, activities directly related to final consumption will increase relatively easily. As a result, the activities in most service sectors will tend to slightly increase. On the other hand, there are slight decreases in mining, general-purpose machinery, production machinery, and power generation, and significant decreases in oil products and coal products. The decreases in oil products and coal products are due to more efficient logistics and decreased inputs in many production sectors.

**Fig. 6:**
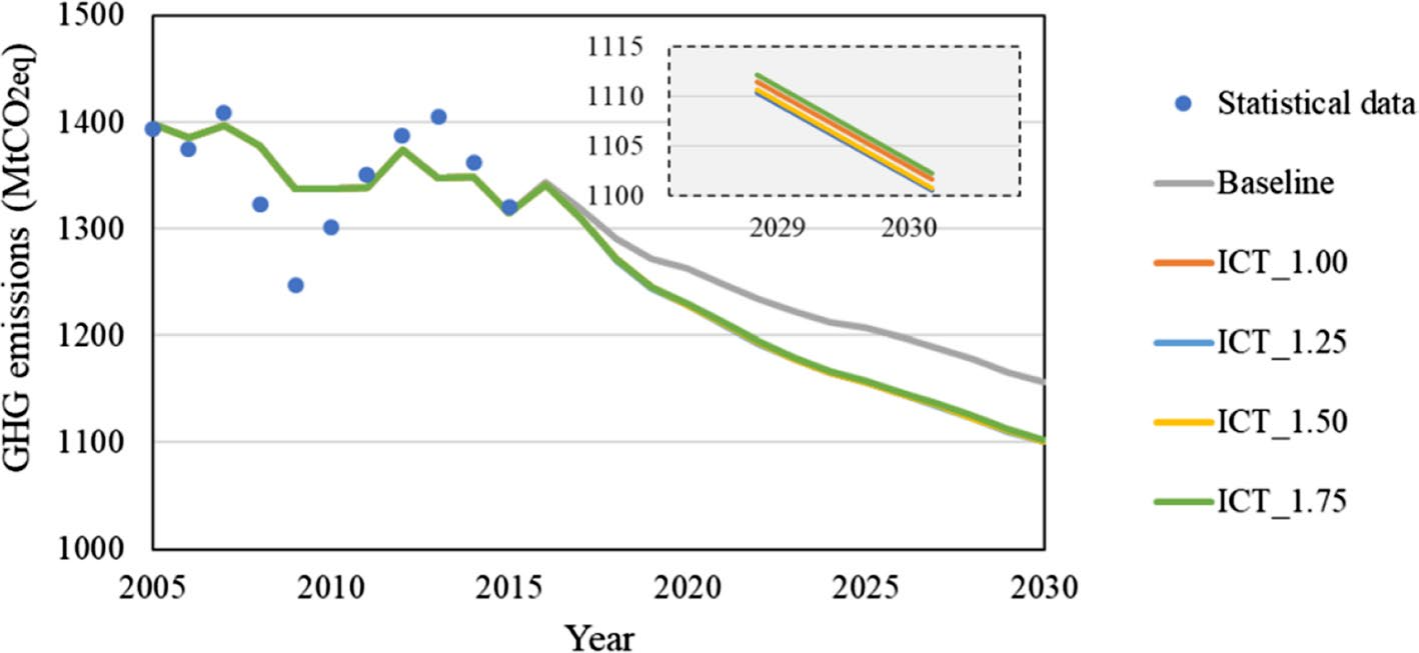
GHG emissions estimation from 2005 to 2030 in each scenario (unit: MtCO_2eq_)

**Fig. 7:**
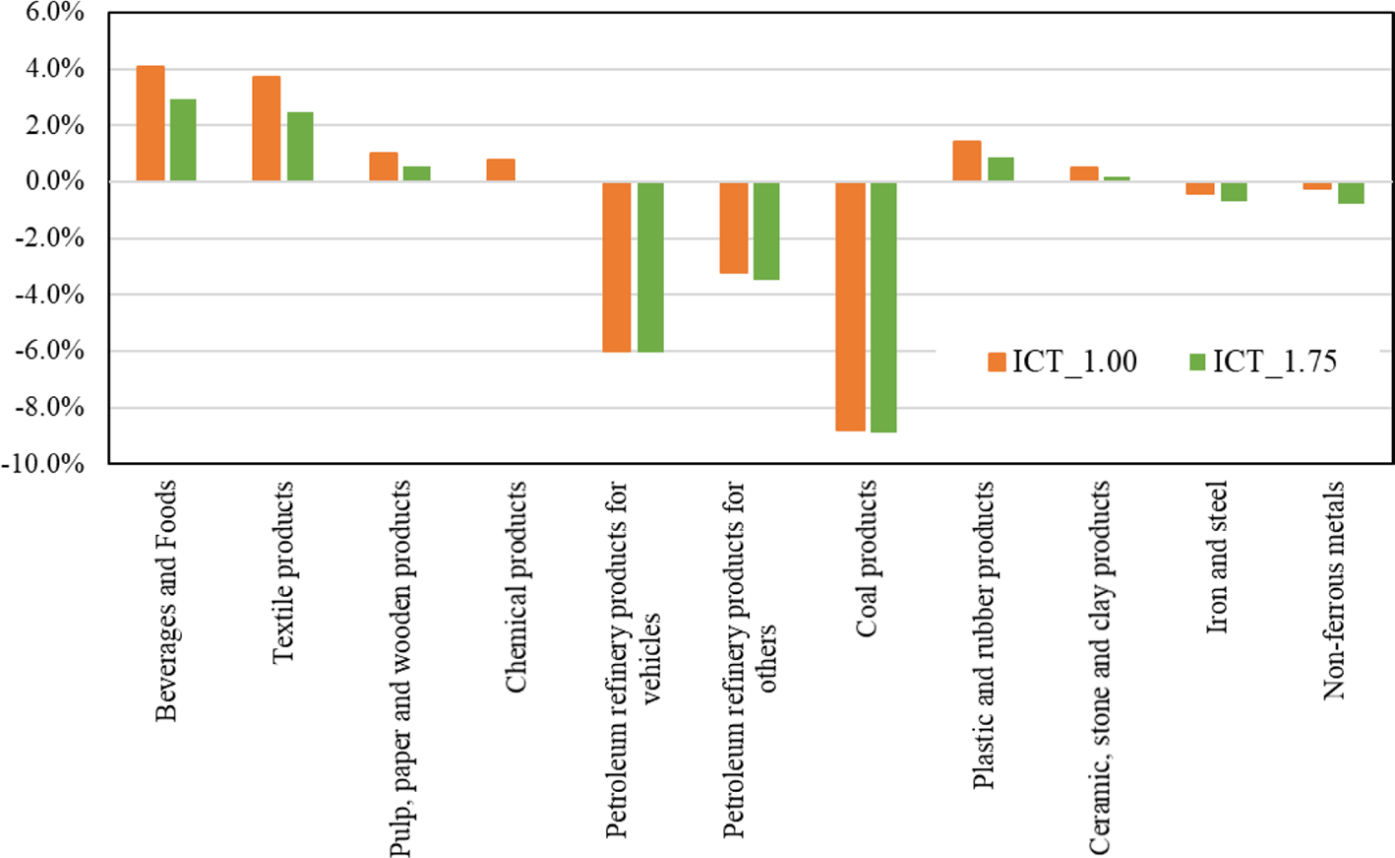
Changes in the demand of materials in 2030 (value in baseline scenario = 1)

Then, regarding GHG emissions, as shown in Fig. 6, GHG emissions decrease from 1400 MtCO_2_eq in 2005 to 1150 MtCO_2_eq in 2030 even in the baseline scenario, as a result of modifying the power supply configuration by the government plan in NDC (MOFA, 2015). Then, in the ICT accelerated scenario, GHG emissions are reduced by 4.7% in 2030 in the ICT_1.00 case compared with the baseline. In addition, in ICT_1.25, ICT_1.50, and ICT_1.75 where service demand for the ICT sector increases by 1.25, 1.50, and 1.75 times, GHG emissions are reduced by 4.8%, 4.8%, and 4.47%, respectively, compared with the baseline. GHG emissions do not significantly change compared with GDP because the power consumption of the ICT sector is a relatively small part of total power consumption in Japan, and the increase in demand for ICT services may affect other inputs and offset them. This means that even when the increase in future power consumption of ICT equipment use is considered in this model, total GHG emissions in 2030 will also be reduced through ICT service usage. For more details, Appendix 1.1 shows the changes in GHG emissions in each sector. Lastly, this paper also briefly analyzes the changes in the demand for materials. If production activities become more efficient due to the advances in ICT services, the amount of materials unnecessarily consumed until now will possibly be saved. Therefore, analyzing the materials will clarify such indirect effects. Figure 7 shows the changes in the demand for each material in 2030 in the ICT accelerated scenario compared with the baseline. The material commodities are shown in shaded cells in Appendix 1.1. Since introducing ICT services will improve the efficiency of logistics and energy saving, the demand for fossil fuels will be greatly reduced. The demand for steel and non-ferrous metals will slightly decrease, which also means that the increased use of ICT will not increase metal consumption. In contrast, demand for food and beverage products and textile products directly related to final consumption will increase over 2%, while demands for others will mostly increase less than 1%.

### Discussion

The results showed that though the spread of ICT will increase the power demand of the ICT sector itself, it will contribute much more to reducing energy demand in other sectors. Total GHG emissions in Japan in 2030 can be reduced up to about 4.8%, with over 1% additional GDP growth, compared with the baseline scenario.

However, this research contains some uncertainties. For example, first, the most basic preconditions in this research are based on the forecast reports in Japan’s Intended Nationally Determined Contribution (INDC) submitted in 2015 (MOFA, 2015), since such macroeconomic data are not updated frequently. The assumed GDP growth rate may be higher than in reality. Second, due to the COVID-19 Pandemic, the world is likely to change significantly in the future. In particular, the COVID-19 Pandemic has triggered a major advance in ICT throughout the world. In Japan, for example, the telework utilization rate is now much higher than in 2019 (Okubo, 2020). Also, the uses of electronic cash (EC) and cashless payment are also increasing rapidly. Furthermore, ICT is expected to drastically spread in areas such as online education and online medical care, which have not been evaluated this time (because their current utilization rates are low). As a result, the GHG reduction effect may be even greater than in the current calculation results in this research. Third, on the other hand, the ICT skill level in the workforce will also affect the speed of ICT popularization. The shortage of personnel sufficiently skilled in cutting-edge technologies is even being felt in Silicon Valley (Metz, 2017), so this shortage is another concern that should be considered.

A scenario analysis like this study aims to show potential future scenarios rather than predict the future correctly and to present ways to move toward a more desirable future. In that sense, more scenario settings may need to be studied in future works, taking into account the above concerns.

### Future issues

Several technical issues are also considered for our future works. First, although the model reflected the increase in additional energy consumption due to ICT introduction, it needs to be further refined in the future because the ratio is set uniformly for all sectors. Second, naturally, in addition to the introduction of such ICT services, the introduction of various advanced technologies such as energy-saving techniques in other sectors and the expansion of renewable energy should also be considered at the same time. However, to clarify the impacts of ICT introduction, in this research, only the acceleration of the ICT level is considered. GHG emissions may decline further considering technological progress in areas other than ICT.

Furthermore, from an environmental aspect, excluding GHG emissions and resource consumption discussed in this research, ICT use may have a wider footprint including pollution associated with mining rare metals, exploiting water resources, and generating electronic waste leading to further pollution of ecosystems. This research focused on the GHG emissions caused by ICT usage and demand for raw materials, since these two factors are our biggest concerns currently. However, other issues are also important for us and are expected to be further researched.

### Values and limitations

With concern regarding increasing ICT traffic globally, this research provided a method to evaluate the future overall impacts of ICT introduction by considering both the increase in energy consumption of ICT equipment itself and corresponding reduction of environmental impact in other sectors. This method can help us analyze whether a given scenario of ICT use will contribute to the realization of a sustainable society. Further, this method can also help us to think out more sustainable ways to use ICT by extending scenarios that are more environmentally and economically friendly and identifying latent problems in ICT development. The results of this research are based solely on the ICT utilization in Japan. The use of ICT and the speed of ICT development, especially the direct effects of ICT use shown in Table 2, may not be the same in each country. Researchers can use this method to evaluate the impacts of ICT use in their own countries on the basis of their countries’ own data.

## Conclusion

Although the rapid spread of information and communications technology (ICT) promotes economic growth and makes daily life more convenient, there is a big concern that ICT equipment use will increase energy consumption. This research adopted a dynamic computable general equilibrium (CGE) model to analyze the future environmental and economic impacts until 2030 brought about by popularization of 36 kinds of ICT services, considering both positive effects of ICT use in non-ICT sectors and negative effects caused by ICT equipment’s operation in the ICT sector.

The model results showed that besides the development of ICT-related sectors, the spread of ICT services, especially some artificial intelligence (AI)-based services, can improve productivity through labor-saving and better matching of supply and demand and contribute to increasing overall GDP. Then, regarding the above concern of the increased energy consumption of ICT equipment, the model results also showed that total greenhouse gas (GHG) emissions will not greatly increase, since the power consumption of ICT services is a relatively small part of overall power consumption, and the increase in service demand for ICT may affect other inputs and offset them. Particularly, efficiency of logistics and manufacturing can greatly reduce the input of oil and coal products, thereby drastically reducing GHG emissions. At the same time, most material demands also decreased on different levels due to the dematerialization brought about by ICT usage. In 2030, compared with the baseline scenario, at least 1% additional GDP growth and 4% GHG emission reduction can be expected by introducing targeted ICT in the ICT accelerated scenario, which also means the feasibility of decoupling the economic and environmental effects of ICT use.

In the future, ICT will surely be more widespread than described here. There are great expectations for digital transformation and automation technologies for agriculture, manufacturing, and service industries. Breakthroughs in productivity by ICT will enable fewer inputs including raw materials and energy and more production and thereby drive greater economic development and environment load reduction. During and after the COVID-19 Pandemic, ICT should help us to achieve an economic recovery without rebounding GHG emissions and build a sustainable society in the next few decades.

## Figures and Tables

**Fig. 8:**
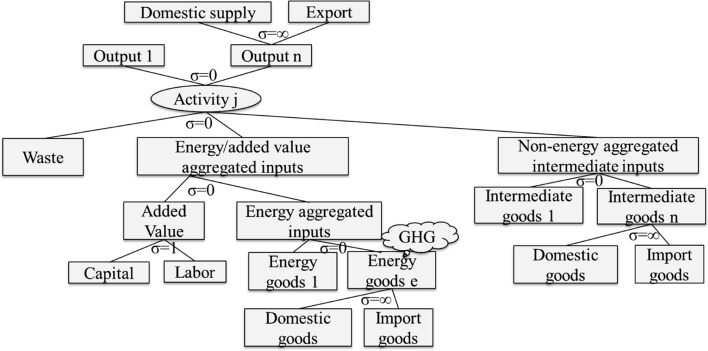
Structure of production activities

**Fig. 9:**
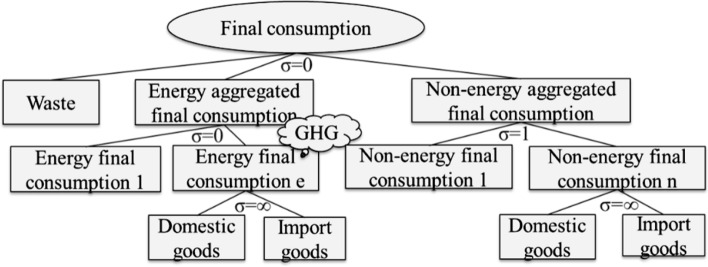
Structure of final consumption

**Fig. 10:**
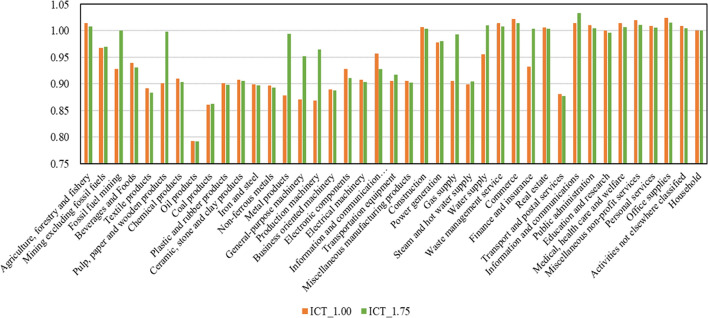
GHG emissions from each sector in ICT accelerated scenario in 2030

**Fig. 11:**
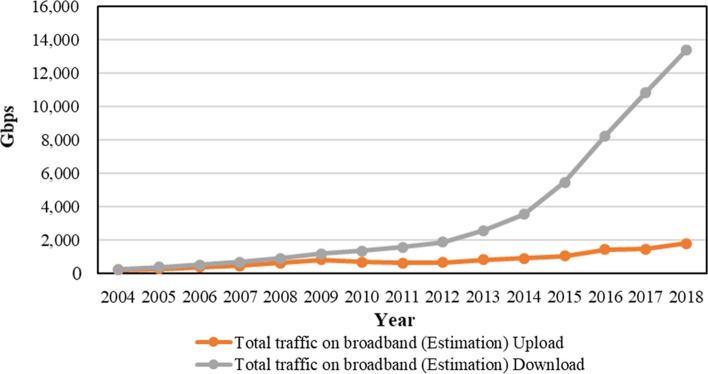
Past communication traffic trends in Japan

**Fig. 12:**
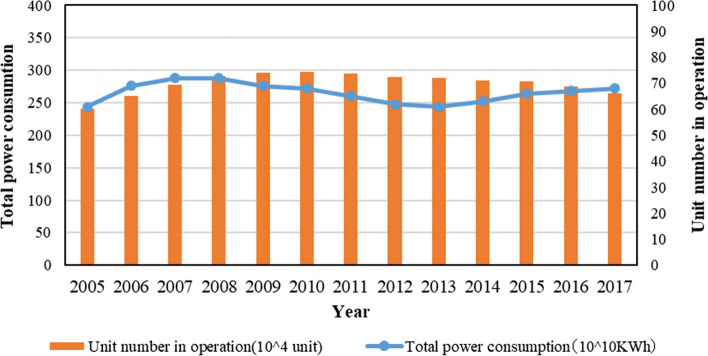
Total power consumption and unit number of servers

## Data Availability

The datasets during and/or analyzed during the current study are available from the corresponding author on reasonable request.

## Appendix 1 Details on the CGE model structure and detailed results

This appendix presents details on the CGE model’s structure and estimated results. Table 3 shows the sector classification in the model, while Figs. 8 and 9 show the nest structure of production activities and final consumption. The key formulas of the model are shown in Table4. Table 5 details the results of GDP and GHG emissions from 2005 to 2030, and Fig. 10 shows GHG emission changes in each sector in the ICT accelerated scenario compared with the baseline scenario in 2030.

**Table 3:** Sectors and commodities in this model

Sector		Commodity	
01	Agriculture, forestry and fishery	01	Agriculture, forestry and fishery
02	Mining excluding fossil fuels	02	Mining excluding fossil fuels
03	Fossil fuel mining	03c	Coal mining
		03o	Crude petroleum
		03 g	Natural gas
04	Beverages and Foods	04	*Beverages and Foods*
05	Textile products	05	*Textile products*
06	Pulp, paper and wooden products	06	*Pulp, paper and wooden products*
07	Chemical products	07	*Chemical products*
08	Oil products	08 m	*Petroleum refinery products for vehicles*
		08o	*Petroleum refinery products for others*
09	Coal products	09	*Coal products*
10	Plastic and rubber products	10	*Plastic and rubber products*
11	Ceramic, stone and clay products	11	*Ceramic, stone, and clay products*
12	Iron and steel	12	*Iron and steel*
13	Non-ferrous metals	13	*Non-ferrous metals*
14	Metal products	14	Metal products
15	General-purpose machinery	15	General-purpose machinery
16	Production machinery	16	Production machinery
17	Business-oriented machinery	17	Business-oriented machinery
18	Electronic components	18	Electronic components
19	Electrical machinery	19	Electrical machinery
20	Information and communication electronics equipment	20	Information and communication electronics equipment
21	Transportation equipment	21	Transportation equipment
22	Miscellaneous manufacturing products	22	Miscellaneous manufacturing products
23	Construction	23	Construction
24	Private power generation	24	Electricity
24n	Nuclear		
24tc	Coal thermal power		
24to	Oil thermal power		
24tg	Gas thermal power		
24 h	Hydro		
24 s	PV		
24w	Wind		
24 g	Geothermal		
24b	Biomass		
25	Gas supply	25	Gas supply
26	Steam and hot water supply	26	Steam and hot water supply
27	Water supply	27	Water supply
28	Waste management service	28	Waste management service
29	Commerce	29	Commerce
30	Finance and insurance	30	Finance and insurance
31	Real estate	31	Real estate
32	Transport and postal services	32	Transport and postal services
33	Information and communications	33	Information and communications
34	Public administration	34	Public administration
35	Education and research	35	Education and research
36	Medical, health care, and welfare	36	Medical, health care, and welfare
37	Miscellaneous non-profit services	37	Miscellaneous non-profit services
38	Personal services	38	Personal services
39	Office supplies	39	Office supplies
40	Activities not elsewhere classified	40	Activities not elsewhere classified

The italic values show material commodities

**Table 4:** Key formulas used in the model

Blocks	Key formulas
Production sectors	$\mathop \sum \limits_{{i = 1}}^{{43}} P_{i} X_{{ij}} + P_{K} K_{j} + P_{L} L_{j} = \mathop \sum \limits_{{i = 1}}^{{43}} P_{i} Y_{{ij}}$
Household sector	$H = P_{K} \mathop \sum \limits_{{j = 1}}^{{49}} K_{j} + P_{L} \mathop \sum \limits_{{j = 1}}^{{49}} L_{j}$
	$$H = \mathop \sum \limits_{{i = 1}}^{{43}} P_{i} \left( {C_{i} + \mathop \sum \limits_{{j = 1}}^{{49}} I_{{ij}} } \right)$$
	$$K_{{j,t + 1}} = \left( {1 - \delta _{j} } \right)K_{{j,t}} + \mathop \sum \limits_{{i = 1}}^{{43}} I_{{ij}}$$
Market equilibrium	$P_{i} \left\{ {\mathop \sum \limits_{{j = 1}}^{{49}} Y_{{ji}} - \left( {\mathop \sum \limits_{{j = 1}}^{{49}} X_{{ij}} + C_{i} + \mathop \sum \limits_{{j = 1}}^{{49}} I_{{ij}} } \right)} \right\} = 0,~$
	$$P_{i} \ge 0~{\text{and}}~\mathop \sum \limits_{{j = 1}}^{{49}} Y_{{ji}} - \left( {\mathop \sum \limits_{{j = 1}}^{{49}} X_{{ij}} + C_{i} + \mathop \sum \limits_{{j = 1}}^{{49}} I_{{ij}} } \right) \ge 0$$
	$$P_{K} \left\{ {K^{*} - \mathop \sum \limits_{{j = 1}}^{{49}} K_{j} } \right\} = 0,~P_{K} \ge 0~{\text{and}}~K^{*} - \mathop \sum \limits_{{j = 1}}^{{49}} K_{j} \ge 0$$
	$$P_{L} \left\{ {L^{*} - \mathop \sum \limits_{{j = 1}}^{{49}} L_{j} } \right\} = 0,~P_{L} \ge 0~{\text{and}}~L^{*} - \mathop \sum \limits_{{j = 1}}^{{49}} L_{j} \ge 0$$

*P:* price, *X:* intermediate inputs, *Y*: output, *K*: capital, *L*: labor, *H*: income (expenditure), *K*^***^: capital endowment, *L*^***^: labor endowment, *C*: final consumption, *I*: investment, *i*: commodities, *j*: sector, $\delta$: depreciation rate

**Table 5:** Detailed results of GDP and GHG emissions from 2005 to 2030

Year	GDP (Trillion Japanese Yen) (constanst 2005)				GHG emission (MtCO2eq)			
	Statistic data	Baseline	ICT_1.00	ICT_1.75	Statistic data	Baseline	ICT_1.00	ICT_1.75
2005	506.1	506.1	506.1	506.1	1393.3	1397.6	1397.6	1397.6
2006	513.3	516.4	516.4	516.4	1374.3	1386.1	1386.1	1386.1
2007	521.8	524.6	524.6	524.6	1409.2	1397.0	1397.0	1397.0
2008	516.1	520.5	520.5	520.5	1323.5	1377.7	1377.7	1377.7
2009	488.1	495.1	495.1	495.1	1247.6	1337.0	1337.0	1337.0
2010	508.6	515.9	515.9	515.9	1301.4	1338.0	1338.0	1338.0
2011	508.0	508.5	508.5	508.5	1351.1	1338.4	1338.4	1338.4
2012	515.6	517.4	517.4	517.4	1386.9	1373.8	1373.8	1373.8
2013	525.9	524.7	524.7	524.7	1404.6	1347.9	1347.9	1347.9
2014	527.7	522.5	522.5	522.5	1361.5	1348.3	1348.3	1348.3
2015	533.5	527.8	527.8	527.8	1320.9	1314.7	1314.7	1314.7
2016	539.0	530.9	531.4	531.2		1344.0	1341.5	1341.3
2017		543.4	545.0	544.4		1319.1	1309.6	1309.6
2018		556.4	559.4	558.2		1291.2	1271.3	1272.0
2019		566.0	570.3	568.6		1272.3	1245.1	1245.7
2020		577.9	583.3	581.1		1262.4	1228.6	1229.8
2021		590.0	596.5	593.9		1247.4	1210.6	1211.9
2022		604.1	611.7	608.6		1233.5	1193.1	1194.3
2023		617.1	625.7	622.2		1222.8	1178.4	1179.6
2024		629.6	639.1	635.2		1212.4	1165.7	1167.0
2025		641.6	651.9	647.7		1207.6	1157.1	1158.2
2026		653.2	664.2	659.7		1198.1	1145.8	1146.9
2027		664.8	676.6	671.7		1189.0	1135.4	1136.4
2028		675.9	688.3	683.1		1178.0	1123.9	1124.7
2029		685.4	698.3	693.0		1165.7	1111.4	1112.2
2030		693.6	707.0	701.4		1156.3	1101.6	1102.2

## Appendix 2 Details on the future estimation on energy consumption of ICT sector

This appendix details the calculation conditions and data used in estimating the power consumption of ICT-related equipment until 2030.

The same as worldwide, total communication traffic on broadband in Japan also increased significantly (Fig. 11) [MICa, 2019]. However, the exponential increase in power consumption of ICT equipment has been constrained by various types of technological progress, such as the increase in the integration rate of semiconductors in accordance with Moore’s law and improvement in power supply efficiency. As an example for servers, Fig. 12 shows the past trends in total power consumption and number of servers based on shipping statistics from the Japan Electronics and Information Technology Industries Association (JEITA). Neither the total number nor power consumption of servers increased much [JEITA, 2016], which means that rated power for each server remained roughly flat.

To catch the change trends of power consumption of ICT equipment itself, we attempted to estimate the total power consumption of ICT equipment until 2030 by estimating the changes in the number of each kind of equipment and the power consumption per unit.

Regarding the former, the change in the number of each kind of equipment by 2030 was estimated on the basis of two kinds of information. One is an investigation into the number of ICT equipment between 2008 and 2018. The other is forecast information for the next 10 years, such as the commercialization of 5G or spread of edge computers. ICT equipment is classified into three sub-sectors: network equipment, data center equipment, and end-user devices. Table

**Table 6:** Main ICT equipment included in this estimation

Intended use		Equipment type
DC	Server	General-purpose computers (main frame)
		Midrange computers
		Personal computers for server use
		External storages
		Miscellaneous external storages
NW	Network equipment	Digital transmission equipment
		Network connection devices
		Base station communication devices
End-user device	Communication equipment	Telephones
		Facsimiles
		Mobile phones
		Public telephones
	PC	Desktop personal computers
		Laptop computers
	Information terminal	Automatic teller machine
		Other banking terminals
		Kiosk terminals
		Handy terminals
		Other terminal units
	IoT	Telemeter/telecontrol
		Detector, transducer, and transmitters
		Distributed control systems
		Miscellaneous monitoring and control system
		Monitoring instruments for environmental quality

6 lists the main ICT equipment considered in this research. The main data source is the statistics in the Preliminary Report on Indices of Industrial Production from the Ministry of Economy, Trade and Industry (METI, 2019). For some kinds of equipment, statistics for imported goods come from the Communication and Information Network Association of Japan (CIAJ, 2018). The numbers of units in operation were estimated from the numbers of shipments each year, taking into account the useful lifetime of each equipment. The main change trends for the next 10 years considered in this estimation include the following:

- Servers are expected to increase production to meet cloud and edge computing demands.
- Data centers are projected to grow at an annual rate of 14.4% until 2024 (MIC 2018), so servers are also expected to grow at the same rate.
- Digital transmission equipment and network connection equipment peaked in 2014 with the transition to new nodes of telecommunication carriers, and production volume is currently declining. Thus, the equipment will peak in 2025 in line with the transition from the PSTN to IP systems (NTT, 2010).
- Changes in smartphone utilization are assumed to fluctuate almost in proportion to changes in the population.
- Laptop utilization is projected to remain flat.
- Other information terminals such as IoT will grow at an annual rate of 5% (MICb, 2019).

Then, regarding the latter, the energy-saving performance of many devices has improved in recent years. As an example, as shown in Fig. 12, even though each server was processing more information, it did not consume more power. However, there is not enough detailed data on the changes in the power consumption per unit of all kinds of ICT equipment. In this research, as a sensitivity analysis, three kinds of assumption are set. One is power consumption per unit does not change until 2030. The second is it will increase by 1% per year from 2020 to 2030. The third is it will increase by 2% per year from 2020 to 2030. Estimation results are shown in Fig. 3 in Sect. 3.4. Power consumption of total ICT equipment in 2030 will increase by 1.2 to 1.5 times that in 2020 under the three assumptions.

Compared with the forecasts in other research, ITU-T reported power consumption of the global ICT sector in 2030 will increase to 1.3 times that in 2015 [L.1470, 2020]. Then, according to the estimation by the Green IT Promotion Council, energy consumption of ICT equipment in Japan in 2030 will increase to about 2 times that in 2020 [JEITA, 2013]. Therefore, a 1 to 2 times increase in power consumption of ICT equipment from 2015 to 2030 can be considered reasonable.
